# Rheological and Flexural Strength Characteristics of Cement Mixtures through the Synergistic Effects of Graphene Oxide and PVA Fibers

**DOI:** 10.3390/polym16040482

**Published:** 2024-02-08

**Authors:** Byoung Hooi Cho, Dong Wook Choi, Mi Hwan Park

**Affiliations:** Department of Civil Engineering, Sangmyung University, 31 Sangmyeongdae-gil, Dongnam-gu, Cheonan-si 31066, Republic of Korea; cloverlove46@daum.net (D.W.C.); mary258@naver.com (M.H.P.)

**Keywords:** graphene oxide (GO), polyvinyl alcohol (PVA) fiber, cement paste, rheological properties, flexural behavior, digital image correlation (DIC)

## Abstract

This study investigates the synergistic effects of incorporating graphene oxide (GO) and polyvinyl alcohol (PVA) fibers into cement paste mixtures, aiming to modify their rheological properties and flexural behaviors with resistance to crack formation. The relationship between static yield stress and critical shear strain was examined in ten cement paste mixtures with varying concentrations of 6 mm and 12 mm PVA fibers and 0.05% GO. Additionally, viscosity analyses were performed. For the specimens fabricated from these mixtures, flexural strength tests were conducted using the Digital Image Correlation (DIC) technique for precise strain analysis under load history. The results indicated a significant increase in static yield stress, viscosity, and critical shear strain due to the combined addition of GO and PVA fibers, more so than when added individually. Notably, in PVA fiber-reinforced cement mixtures, the integration of GO increased the crack initiation load by up to 23% and enhanced pre-crack strain by 30 to 50%, demonstrating a notable delay in crack initiation and a reduction in crack propagation. Microstructural analyses using Scanning Electron Microscopy (SEM) and Energy Dispersive X-ray Spectroscopy (EDS) revealed a concentrated presence of GO around and on the PVA fibers. This promotes increased C-S-H gel formation, resulting in a denser microstructure. Additionally, GO effectively interacts with PVA fibers, enhancing the adherence of hydration products at their interface.

## 1. Introduction

Cementitious composites are widely used in the modern construction industry as a material with high elastic modulus and compressive strength, but relatively low tensile and flexural strength, and it exhibits brittle behavior at failure. To overcome these disadvantages, extensive research has been conducted to improve mechanical characteristics through the incorporation of various types of fibers such as steel, basalt, polyvinyl alcohol (PVA), and polypropylene (PP). However, these fiber-reinforced cement mixtures are used to suppress the progression of relatively large-scale cracks (macro cracks); hence, the cracks are of a few millimeters to centimeters in size. Cracks in cement composites, on the other hand, start from micro cracks during load application and progress to a visible scale. Therefore, research is actively being conducted to control these initial micro cracks effectively by incorporating carbon-based nanomaterials, such as carbon nanotubes (CNT), graphene, and graphene oxide (GO), etc.

Among the carbon-based nanomaterials, GO is a two-dimensional form of a single layer of carbon with high strength and toughness, and it has functional oxygen groups like hydroxyl (-OH), carboxyl (-COOH), amine (-NH2), carbonyl (C=O), and epoxy (C-O-C) [1,2]. These oxygen groups are known to accelerate the cement hydration process and contribute to the strength development of the hydrates [3,4]. Previous studies have shown that incorporating 0.02 to 0.08% of GO by weight of cement in cement paste and mortar mixtures increases compressive and flexural strength by 29 to 46% and 26 to 70%, respectively, and tensile strength by up to 40% with 0.03 to 0.06% GO [5,6]. However, GO produced by the traditional chemical Hummer method is expensive, making its large-scale application in the construction industry economically challenging. A mechanochemical method, using ball milling to significantly reduce the production cost of GO, was recently developed [7]. GO, produced by the method referred to as the ‘Garmor method’ in this study and costing less than 40 USD per kilogram, has potential for large-scale application in cement-based construction materials [8]. Preliminary research confirms that cement paste and mortar mixtures containing this GO show positive strength enhancements [9,10,11], especially in terms of resistance to initial micro crack formation and progression, enhancing fatigue performance under repeated loads [12]. However, the large specific surface area of GO [13] and the presence of functionalized oxygen groups on its surface mean that incorporating GO can increase the viscosity of cement mixtures and reduce their workability performance [8,11]. This presents a challenge for the use of GO in the construction field.

PVA fiber, a hydrophilic synthetic fiber, is widely used to enhance toughness and resistance of crack propagation in cement-based construction materials [14]. Not only does the high tensile strength of the fiber itself contribute to this effect, but its hydrophilic oxygen groups on the surface enable strong bonding with cement hydrates, improving the mechanical performance of the hydrated cement mixture [15,16]. Nonetheless, PVA fibers significantly influence the rheological properties essential for handling cement mixtures [17,18]. Their addition notably reduces the workability of the mixtures [19,20], which is crucial for ensuring straightforward application and achieving consistent quality in cementitious composites.

In this study, the synergistic incorporation of GO and PVA fibers into cement paste is emphasized. This strategy is underpinned by the objective of simultaneously controlling microcracks and macrocracks within the cement matrix. GO is anticipated to effectively control the initiation and propagation of microcracks. Conversely, PVA fibers are employed to manage macrocracks, contributing to the prevention of sudden collapse under continuous stress conditions. Furthermore, the potential synergistic interaction between GO and PVA fibers is explored. It is hypothesized that this interaction, occurring within the cement matrix, could significantly influence the composite’s overall performance. This includes aspects such as crack resistance, workability, and mechanical properties. The research not only delves into the individual contributions of GO and PVA fibers but also investigates the combined effects and potential synergies arising from their interaction. Therefore, in this study, flexural strength and rheology tests were conducted on cement paste mixtures reinforced by GO and PVA fibers. The combined effect of GO and PVA fibers on flexural behavior was discussed by analyzing their influence on crack initiation and propagation to the macro scale. Additionally, the effects of GO and PVA fibers on the rheological characteristics of the cement paste mixtures were experimentally studied. The Digital Image Correlation (DIC) technique was employed to discuss the flexural behavior of the mixtures. Scanning Electron Microscopy (SEM) and Energy-Dispersive X-ray Spectroscopy (EDS) were also conducted for microscopic analysis.

## 2. Experimental Program

### 2.1. Materials and Mix Proportion

**Figure 1:**
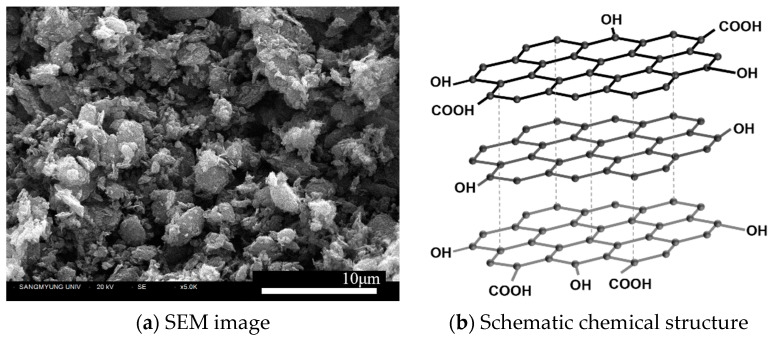
GO produced by Garmor method.

**Figure 2:**
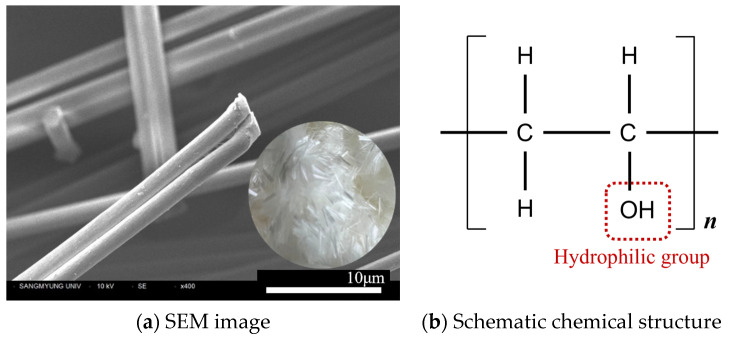
PVA fiber.

Cement paste mixtures with a water-to-cement ratio (w/c) of 0.5 were used. The chemical composition of the Type I Ordinary Portland Cement (OPC) used in this study is as shown in Table 1. The traditional Hummers method for producing GO involves a chemical process using oxidizing agents, utilizing strong oxidizers such as KMnO_4_, H_2_SO_4_, and H_2_O_2_. In contrast, the GO utilized in this study is synthesized through an economical ball-milling technique. This method involves a harmless oxidizing agent and is cost-efficient, with a price below US$40/kg, which is particularly advantageous for incorporation into cement matrices. The synthesis is characterized by a mechanochemical process where graphite powder is finely milled with the oxidizing agent in a controlled environment. This approach is designed to optimize shearing forces while simultaneously reducing collision forces. As a result, the oxidation of graphite occurs predominantly along the edges, and the layers are exfoliated in a manner akin to unzipping. The outcome of this process is a GO with only a few layers. Figure 1a and Figure 2b show the SEM image of GO produced by Garmor method and the multi-layered structure with functionalized oxygen groups such as hydroxyl and carboxyl formed along the periphery of the flake structure, respectively. The PVA fibers used in this study are synthetic monofilament fibers with an absorption rate of less than 5%, as presented in Figure 2. Since PVA fibers exhibit hydrophilicity due to the presence of hydroxyl groups (-OH), they are characterized by excellent bonding performance within the hydrated cement matrix.

The chemical and physical properties of GO and PVA fibers utilized in this study are presented in Table 2. The oxygen content in GO is between 5 and 10%, which is relatively low compared to the 40–50% typically observed in GO produced via Hummers method [22,23]. The average particle size of GO is approximately 450 nm in diameter. Considering that the thickness of a single graphene sheet is about 0.7–1.0 nm, it can be inferred that the GO consists of flakes with fewer than 10 layers, culminating in a maximum thickness of around 10 nm. Furthermore, PVA fibers of two different lengths were used, both with an elongation of less than 8%, and lengths of 6.0 mm and 12.0 mm. The fibers have a diameter of less than 26 μm, with aspect ratios of 231 and 462, respectively. They exhibit a tensile strength of 1200 MPa and a modulus of elasticity of 24.5 GPa.

The mix proportions and the identifiers (IDs) for each cement paste mixture containing PVA fibers and GO are detailed in Table 3. The w/c was uniformly set at 0.5 across all mixtures. PVA fibers in lengths of 6.0 mm and 12.0 mm were incorporated at volume percentages (vol%) of 1% and 2%. GO was mixed at a weight percentage (wt%) of 0.05% relative to the cement, resulting in a total of 10 different mixtures. Previous research indicates that the addition of 0.05% GO optimally enhances the mechanical properties of cement composites [10]. The GO was dry mixed with the cement. To ensure uniform distribution of GO powder, an initial mixing with a small amount of cement was conducted before the remaining cement was incorporated, employing a dry mixing method. This approach was preferred over the wet mixing method, which involves an ultrasonication process, as it is more economical. Despite a slight decrease in GO dispersion, previous studies [11,24] report that the dry mixing method achieves properties comparable to the wet mixing method in terms of rheological and mechanical characteristics. For the ten mixture IDs, 6 specimens each for flexural strength testing (40 mm × 40 mm × 200 mm) were fabricated. The specimens were subsequently cured in a water bath for 28 days. Rheological tests were also performed for all mixtures.

### 2.2. Test Procedure

#### 2.2.1. Rheological Test

**Figure 3:**
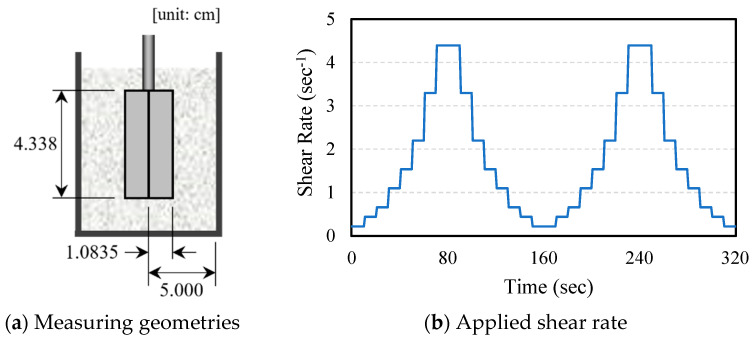
Rheological test.

In this study, the rheological characteristics of ten mixtures were quantitatively evaluated using a Brookfield DV2T Viscometer manufactured by AMETEK Brookfield, Middleborough, MA, USA. A vane spindle was employed, as depicted in Figure 3a. The changes in shear stress were measured over two cycles of increasing and decreasing shear rates, as shown in Figure 3b, to ascertain the viscosity characteristics. Additionally, the yield stress values for each mixture were determined by rotating the vane spindle at a low speed of 0.1 RPM. All tests were conducted at a laboratory temperature of 24 °C and a relative humidity (RH) of 40%.

#### 2.2.2. Flexural Strength Test

To evaluate the flexural strength and behavior of the ten cement mixtures incorporating PVA fibers and GO, experiments were conducted using the three-point bending test method in accordance with ASTM C293 [25]. For the tests, a Universal Testing Machine (UTM) (HD-201 manufactured by Hyundai Precision Industry Co., Ltd., Seoul, Republic of Korea) was employed, which applied the load at a rate of 0.005 mm/min. To precisely measure the fracture load, a dedicated load cell (DBHS-1t) was used to record the load history up to the point of fracture. Concurrently, the displacement history was captured using a DIC system. The collected data were integrated to produce the load-displacement history. The span length (*L*) for the specimens was set at 150 mm, and the flexural strength (*σ*) was calculated using the maximum load at fracture using following formula.

(1) $$\sigma =\frac{3FL}{2bd^{2}}$$

where the definitions were as follows:

*F*: Load at fracture (N)

*L*: Length of support span (mm)

*b*: width of specimen (mm)

*d*: thickness of specimen (mm)

#### 2.2.3. Digital Image Correlation (DIC)

The DIC technique, which originated in the 1980s [26,27], has become a prominent method for measuring strain using digital images [28,29,30]. It evaluates displacement and strain by analyzing images of a specimen’s surface before and after deformation. Particularly beneficial for studying materials like cement concrete, DIC tracks changes in strain distribution and potential crack formation during loading. The technique involves comparing subsets of pixels from pre- and post-deformation images to calculate deformation [31]. Each pixel’s unique grayscale value aids in accurately tracking these subsets.

**Figure 4:**
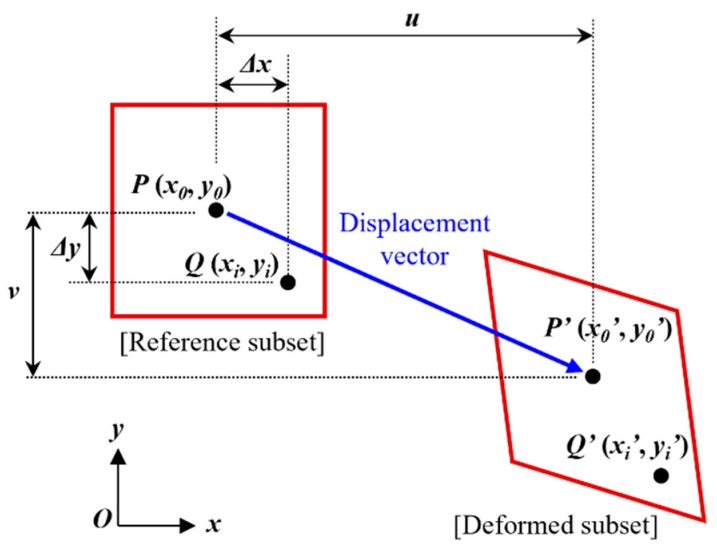
Subset before and after deformation.

**Figure 5:**
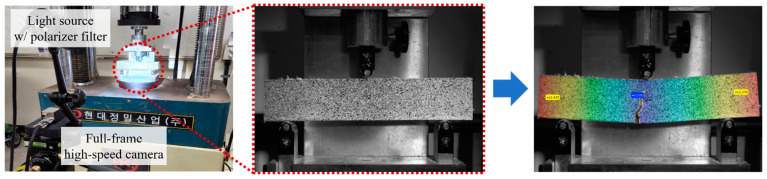
DIC setup and testing example.

In this study, DIC was utilized to assess the flexural behavior of cement paste specimens. To enhance visibility, a contrasting pattern was applied to the specimens, and images were captured using a charge-coupled device (CCD) camera before and after deformation. Figure 4 illustrates the DIC method’s principle, showing how displacement and strain are calculated by matching pixel subsets between reference and deformed images. Figure 5 demonstrates the DIC setup and provides an example of image analysis during testing. The UTM applied the load while the DIC system measured strain and displacement, capturing images at a rate of 20 Hz. For image capture and analysis, the ‘ARAMIS MC 2D’ model, ‘GOM Snap 2D’ software 2020, and ‘GOM Correlate’ software 2020 were used, enabling researchers to analyze displacement and strain at specific locations post-experiment.

#### 2.2.4. Microscopic Analysis

SEM and EDS analyses were conducted to elucidate the microstructural characteristics of selected cement composite samples, which included GO and PVA fibers. For assessing surface morphology, CTRL and GO specimens were meticulously prepared. Additionally, a series of specimens designated as P6-1, P12-1, P6-1-GO, and P12-1-GO were prepared, with an emphasis on capturing the fractured morphology and surface characteristics of the PVA fibers. The SEM analysis was performed using a SNE-3000MS manufactured by SEC, Seoul, Republic of Korea, operated at an accelerating voltage of 10 kV. Furthermore, EDS analysis was specifically carried out on the P12-1-GO specimen, employing a SU5000 SEM system equipped with an EDS detector, also operated at 10 kV.

## 3. Results and Discussion

### 3.1. Rheological Characteristics

#### 3.1.1. Static Yield Stress—Critical Shear Strain

**Figure 6:**
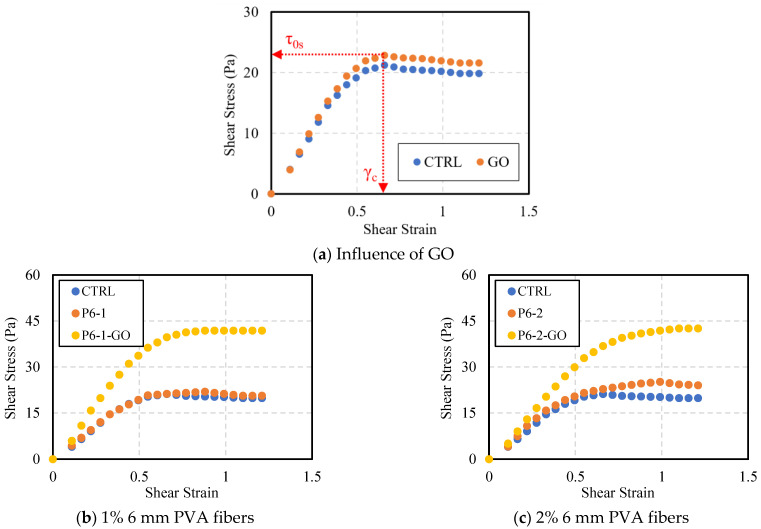

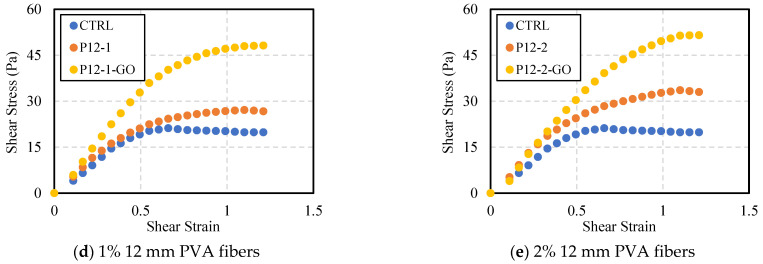
Static yield stress–critical shear strain analysis for additions of GO and PVA fibers.

Figure 6 shows the static yield stress (τ_0s_) and the corresponding critical shear strain (γ_c_) for the ten different cement mixtures. Static yield stress refers to the minimum stress level required for a cement mixture to overcome its internal structure and initiate flow. Critical shear strain denotes the degree of deformation at which the internal structure of the mixture begins to collapse under the static yield stress, thereby inducing flow. Thus, lower values of τ_0s_ and γ_c_ indicate easier initiation of flow. In the graphical representation of shear strain versus shear stress, an initial increase in shear strain under low shear rate conditions results in a corresponding rise in data points. Subsequently, a distinct inflection point is observed where the data points begin to decrease with further increases in shear strain. This particular point is indicative of the static yield stress and the critical shear strain. As observed in Figure 6a, the cement paste mixture without any additives (CTRL) exhibited initial flow at the stress of 21.20 Pa with the strain of 0.66. In contrast, the mixtures with GO showed 7.6% higher static yield stress of 22.82 Pa than CTRL, with the same strain value of 0.66. Figure 6b–e displays the τ_0s_ and γ_c_ values for the mixtures containing PVA fibers and PVA fibers + GO compared to CTRL. For the P6-1 mixture, τ_0s_ was similar to CTRL at 21.94 Pa, but a higher γ_c_ of 0.83 was recorded. P6-2 exhibited an even higher γ_c_ of 0.99, with τ_0s_ at 25.16 Pa, about 18.7% higher than CTRL. Remarkably, the incorporation of GO into PVA fiber reinforced cement mixtures significantly increased τ_0s_. P6-1-GO, compared to P6-1, showed a 91% higher τ_0s_ of 41.87 Pa, with the unchanged strain value of 0.88. For P6-2-GO, τ_0s_ rose by about 69% to 42.59 Pa compared to P6-2, with a slightly increased strain of 1.10. Similarly, mixtures with 12 mm PVA fibers also showed a moderate increase in τ_0s_ due to the PVA fiber addition, with a substantial rise upon further addition of GO. However, the critical shear strain changes were minimal, indicating that, while PVA fiber incorporation significantly increased γ_c_ compared to CTRL, the additional of GO had a negligible impact on the γ_c_. Additionally, although P6-2 has half the fiber length of P12-1 but about four times the number of strands, the similar static yield stress–critical shear strain curves suggest that fiber length plays a more significant role than the number of strands for the rheological characteristics of the cement mixtures.

#### 3.1.2. Shear Rate—Shear Stress

**Figure 7:**
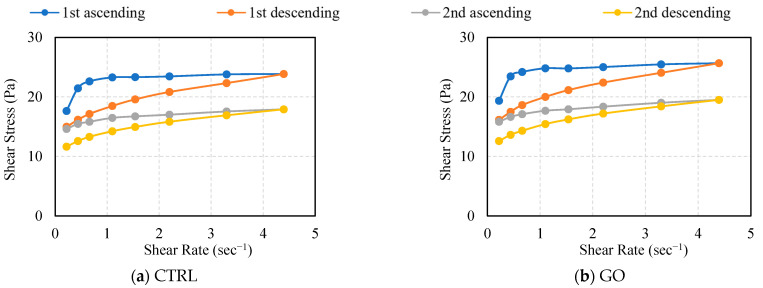

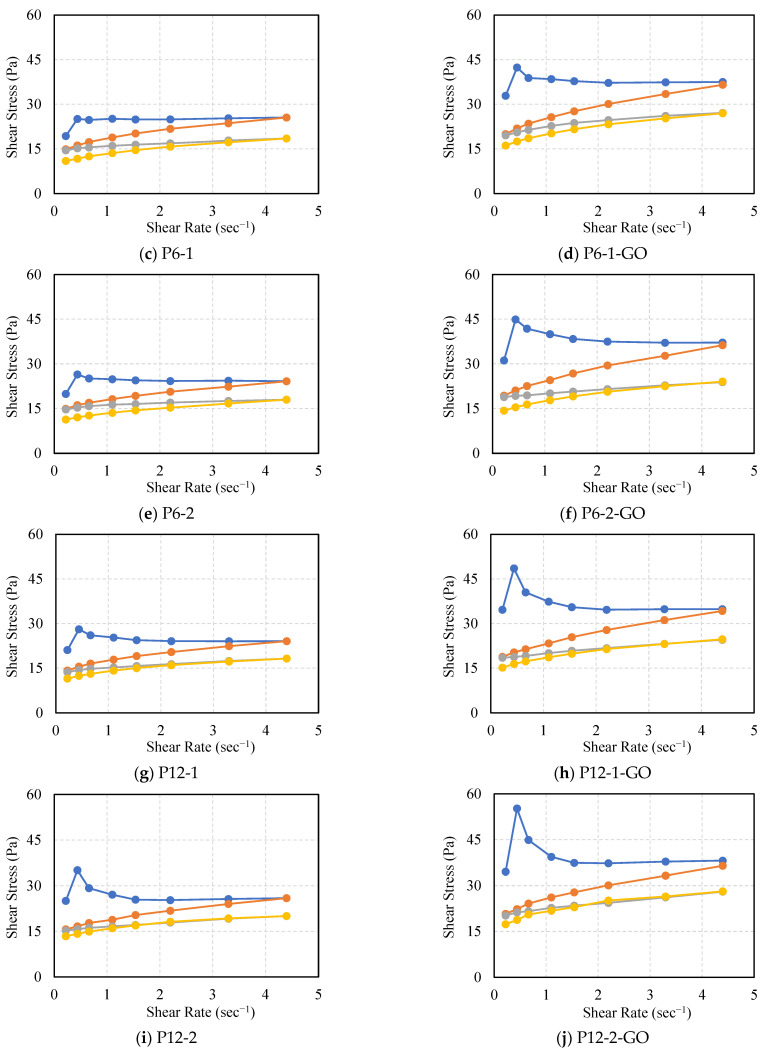
Shear stress–shear strain analysis for additions of GO and PVA fibers.

Figure 7 displays the flow curves, which plot shear stress against shear rate, for the cement mixtures with various combinations of PVA fibers and GO. The curves are divided into four distinct regions, representing two cycles each of increasing (i.e., 1st and 2nd ascending) and decreasing (i.e., 1st and 2nd descending) shear rates. The ratio of shear stress to shear rate is indicative of viscosity, allowing these graphs to facilitate a comparison of relative viscosity among the mixtures, based on the shear stress values. Consequently, In the comparison between CTRL and GO, the GO mixture exhibits consistently higher viscosity across all cycles. This observation is in line with previous studies that suggest the addition of GO decrease fluidity and workability of cementitious mixtures [8,11]. For the mixtures incorporating PVA fibers, an increase in viscosity is also noted upon the addition of GO. Nevertheless, there are noticeable variations in viscosity across the four stages of shear rate changes. In the 1st ascending phase, mixtures with longer and more abundant PVA fibers show a sharp rise in viscosity at lower shear rates, which then levels off as the shear rate continues to increase. The inclusion of GO in the mixtures results in a markedly higher initial viscosity during this phase, particularly in the mixtures with longer and greater amounts of PVA fibers, highlighting the pronounced effect of GO on early-stage flow viscosity. As the shear rate continued to increase, a steady level of viscosity was maintained. Notably, while cement mixtures with only PVA fibers demonstrate minimal viscosity variations with changing shear rates, the mixtures containing both PVA fibers and GO show more pronounced fluctuations in viscosity. This suggests that the combined use of PVA fibers and GO has a more substantial effect on the flow properties of the mixtures than when each is used separately. This effect may be attributed to the interaction between PVA fibers and the cement matrix, which is likely influenced by the presence of GO in the fresh cement mixtures.

### 3.2. Flexural Strength Characteristics

**Figure 8:**
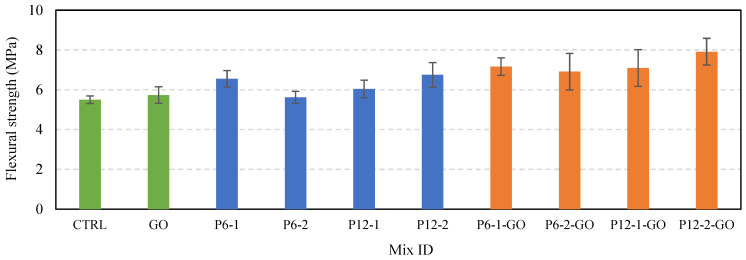
Average flexural strength for the mixtures.

Figure 8 illustrates the 28-day flexural strength results and their deviations for the ten different mixtures. The flexural strengths were calculated based on the first load peak value that caused the initial cracking in the specimens. The control specimen (CTRL) with no additives, and the specimen with 0.05% GO mixed in (GO) measured average flexural strengths of 5.49 MPa and 5.73 MPa, respectively. Although the GO specimen showed a slightly higher strength value, the difference was within the range of deviation, making it challenging to conclusively attribute this increase to the effect of GO. In contrast, the specimens mixed with PVA fibers but without GO showed the values ranging from 5.62 MPa to 6.75 MPa, while those mixed with GO recorded the strengths in the range of 6.91 MPa to 7.91 MPa. Thus, it was observed that the addition of 0.05% GO in the PVA fiber-reinforced cement mixture meaningfully enhanced flexural strength in all cases. It is considered that the specimens with 1% 6 mm PVA fibers (P6-1) and 2% 12 mm fibers (P12-2) have a similar number of fibers, albeit of different lengths. For these mixtures, similar levels of flexural strength were measured without GO; however, the inclusion of GO resulted in P12-2-GO showing an approximate 10.5% increase in average flexural strength compared to the P6-1-GO. This suggests that GO may influence the adhesion and pull-out resistance performance of PVA fibers in the hydrated cement composite.

**Figure 9:**
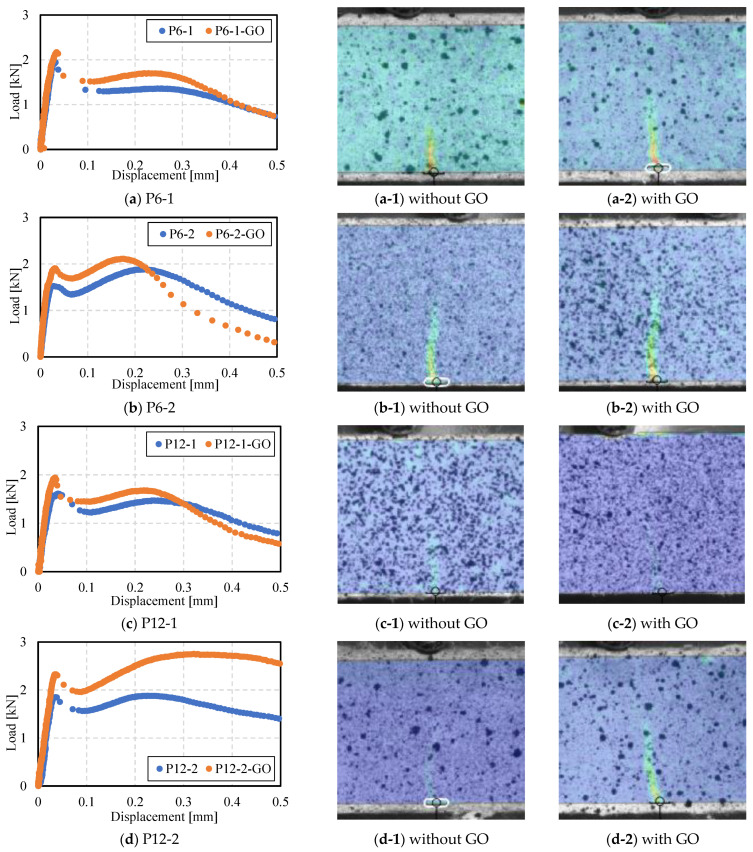
Load–displacement curves for flexural strength tests and DIC analysis.

**Figure 10:**
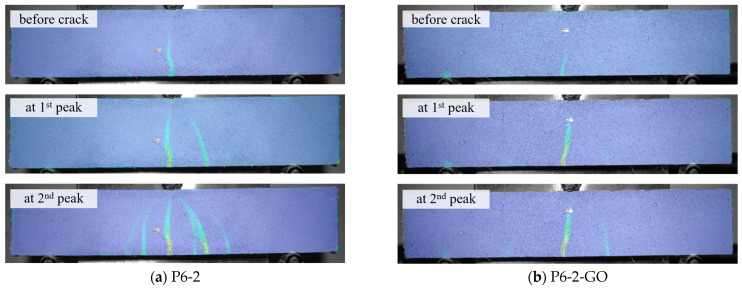
Example of crack propagation in response to loading process with and without GO.

Figure 9 presents the load-displacement curves and the distribution contours of principal strains near the impending crack locations, analyzed by DIC at the first load peak point (i.e., just before crack initiation), for eight mixtures incorporating PVA fibers and GO. CTRL and GO specimens are excluded in this figure. The x-axis represents the mid-span deflection of the specimen, while the y-axis shows the load applied at the center of the specimen. For each mixture, the load-displacement curve of the specimen that is closest to the average of the maximum load values, measured from six samples, is represented. This demonstrates the softening behavior observed in all specimens. Upon reaching the first load peak, crack initiation is observed, followed by a decrease in load as further deformation occurs. This leads to a second load peak and a subsequent gradual decrease. The time interval between each data point on the graph corresponds to the DIC analysis speed of 1/20th of a second. Therefore, a wider interval between data points indicates a more rapid increase in displacement and a faster decrease in load. For example, the P6-1 specimen exhibited a rapid increase in displacement (i.e., brittle behavior) after the initial crack, whereas P6-1-GO showed a relatively slower rate of displacement increase after cracking. This difference is attributed to the influence of GO, suggesting that it partially resisted the pull-out of the 6 mm PVA fibers from the cement hydrates. This effect was less pronounced in specimens with 1% 12 mm PVA fibers. It was noted that both the first load peak, which causes initial cracking, and the second load peak, resulting from the role of PVA fibers, increased in value with the addition of GO. Furthermore, the mid-span deflection corresponding to the second load peak was observed to be smaller with the inclusion of GO. Additionally, in Figure 9(a-1)–(d-2), which represents the moments just before crack initiation, the addition of GO resulted in a broader distribution of increased strain, extending to a higher internal area of the specimens. This suggests that GO may delay the onset of microcracking. Furthermore, the presence of a higher strain concentration may lead to a decreased tendency for developing multiple cracks, as illustrated in Figure 10. This point is especially pertinent for the practical application of these composites in real structures, suggesting that a combination of GO and PVA fibers might result in fewer cracks compared to using PVA fibers alone. However, there is a possibility that these composites might exhibit a more abrupt failure, in contrast to the more gradual failure observed in samples containing only PVA fibers. This situation necessitates a careful consideration of the trade-off between improving durability by minimizing crack formation and ensuring the safety and predictability of the material’s behavior. The challenge involves identifying an optimal mixture of materials that not only enhances structural integrity but also guarantees a predictable and safe failure mode, underscoring the complex process of optimizing material compositions for structural applications.

Table 4 presents the values of the first load peak, which causes the initial crack, along with the corresponding mid-span deflections, and the second load peak with its respective deflections for the ten cement mixtures cured for 28 days. Additionally, the back-calculated elastic modulus (*E*) computed using the first load peak values and the corresponding deflections according to the following equation are included. The modulus of elasticity values is represented as the average of six specimens for each mixture.

(2) $$E=\frac{FL^{3}}{48I\delta_{max}}$$

where the definitions are as follows:

*F*: Load at fracture (kN)

*L*: Length of support span (mm)

*I*: Moment of inertia of cross section (mm^4^)

*δ_max_*: Maximum deflection at facture (mm)

Despite variations in the length and amount of PVA fibers used, the addition of GO to PVA fiber-reinforced cement composites resulted in an approximate 9% to 23% increase in the load at the first peak (1st peak), which caused the initial crack. Additionally, a 2% to 24% increase was observed in the second load peak (2nd peak). Conversely, the mid-span deflection corresponding to the 2nd peak load was identified as decreasing. This reduction can be attributed to GO’s role in partially resisting the pull-out of PVA fibers from the interface between cement hydrates and PVA fibers after cracking. Despite the increased load at the 1st peak, the deflection values did not show significant changes. Consequently, the back-calculated modulus of elasticity values exhibited an increase ranging from approximately 7% to 39% across the mixtures due to the addition of GO. This suggests that GO potentially delays the initiation and propagation of microcracks in the cement matrix under flexural stress. Similarly, it is inferred that the addition of GO can enhance the toughness of PVA fiber-reinforced cement composites. For the P6-1 mixture, while the increase in toughness (i.e., area under the load-displacement curve) due to the addition of GO was minimal at a deflection of three times the maximum at the first load peak (i.e., 3δ_max_, or 0.1 mm), a notable increase in toughness was observed in other mixtures. Therefore, it is anticipated that incorporating a certain amount of PVA and GO simultaneously could lead to an increase in the toughness of cementitious composites.

### 3.3. Virtual Strain Analysis via DIC

**Figure 11:**
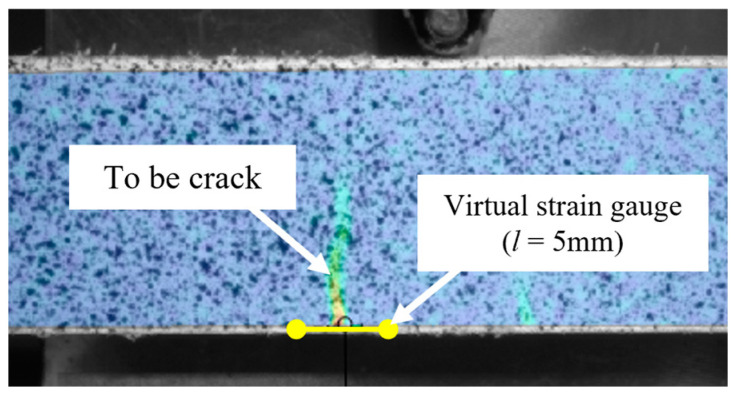
Schematic diagram for virtual strain analysis via DIC.

One significant advantage of using the DIC technique for strain analysis is its ability to determine the precise location where a crack is initiated. This enables the pre-setting of specific regions of interest to capture data relevant to the deformation history of those areas. Utilizing this benefit, this study analyzed the variation in strain at the prospective crack location before and after crack occurrence in the eight different mixtures, each containing PVA fibers and GO. The DIC system facilitates the placement of a virtual strain gauge at any selected location and of any specified length. As depicted in Figure 11, a virtual strain gauge with a total length of 5 mm, extending 2.5 mm on each side from the confirmed crack initiation point, was established. The average strain value (ε_xx_) over this length was computed and is presented in Table 5.

The strain measured at the first peak load represents the deformation just before crack initiation. The smallest strain value (0.00277) was observed in the P6-1 mixture, which did not contain GO, while the largest strain value (0.00426) was recorded for the P12-1 mixture. Notably, mixtures with 2% of 6 mm PVA fibers exhibited an increase in strain compared to those with only 1% fiber content. However, for mixtures with 12 mm PVA fibers, an inverse trend was observed; the strain decreased with a 2% fiber content compared to 1%. It was identified that the addition of GO to all mixtures increased the strain before crack initiation by approximately 30 to 50%. This suggests that GO plays a role in delaying the onset and propagation of microcracks, thus enhancing crack resistance as deformation increases with stress. Conversely, the strain values measured at the second peak load exhibited a different trend. The PVA fiber-reinforced cement composites without GO showed strain values ranging from 0.074 to 0.140, while those with GO ranged from 0.074 to 0.146. This indicates that the strain variation in the cement mixtures due to the addition of GO was relatively minor, approximately –7.5% to 4.5%. This implies that the additional deformation from load increase post-initial crack was minimal, suggesting that GO contributed to resisting further crack propagation. This resistance to crack expansion post-crack initiation is attributed to the pull-out resistance provided by GO between the PVA fiber surfaces and cement hydration products.

**Figure 12:**
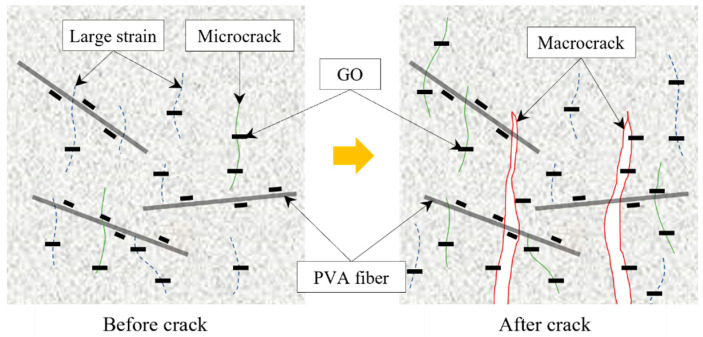
Roles of PVA fibers and GO before and after crack occurrence.

Based on the flexural strength testing and DIC analysis of the cement composites infused with PVA fibers and GO, the roles of these PVA fibers and GO before and after crack initiation are conceptually depicted in Figure 12. As the load increases and stress intensifies, areas of increased strain (indicated by dashed lines) emerge irregularly within the hydrated cement matrix, which can eventually evolve into microcracks (depicted as solid lines). At this juncture, the presence of GO dispersed throughout these areas may delay the initiation and progression of microcracks due to its bridging effect within the microstructure. Upon the occurrence of a crack, it is braced by the PVA fibers, and as the load further increases, the crack widens, causing the PVA fibers to either be pulled out or break, which leads to a loss of load-bearing capacity and ultimately to failure. DIC analysis indicates that the addition of GO to the cement mixtures can comparatively reduce the propagation of cracks. This effect may be attributed to GO enhancing the bond between PVA fibers and the cement hydration products at the interface, thus bolstering the resistance to pull-out forces exerted by the hydrated cement matrix.

### 3.4. Microscopic Analysis

**Figure 13:**
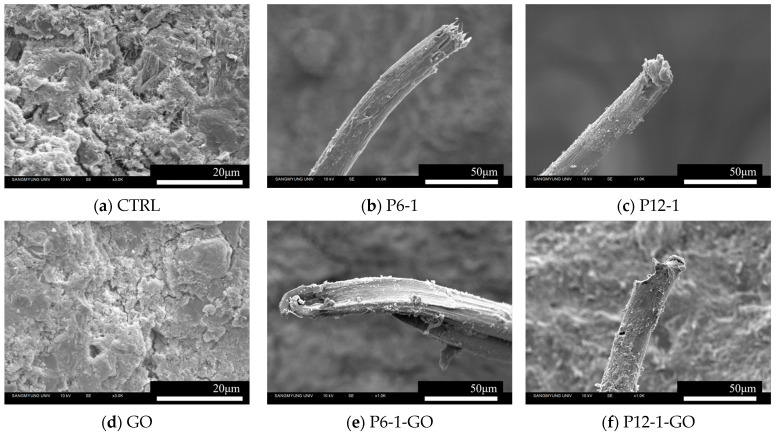
SEM images.

The synergistic effects of incorporating PVA fibers and GO into cement mixtures have been explored through rheological characteristics and flexural strength tests. Subsequent to these tests, a microscopic morphological analysis was conducted using SEM and EDS to further discuss the combined effects. It is widely recognized that the presence of functionalized oxygen groups, particularly carboxyl (–COOH) and hydroxyl (–OH), on GO significantly contributes to the hydration process of cement [9]. These groups facilitate the nucleation or seeding effect, thereby promoting the formation of more calcium silicate hydrate (C–S–H) gel, which is crucial in defining the mechanical properties of hydrated cement composites. SEM images, as depicted in Figure 13a,d, show the microstructures of CTRL and GO mixtures, respectively. The inclusion of GO in the cement mixture results in a denser microstructure. Figure 13b,c displays the fractured PVA fibers in the P6-1 and P12-1 mixtures, while Figure 13e,f reveals the fractured fibers in the P6-1-GO and P12-1-GO mixtures, respectively. In the GO-containing cement mixtures, the PVA fibers exhibit a rougher texture upon fracturing, and a greater quantity of hydration products adhering to the fiber surface is observed. This suggests that GO enhances the adhesive interaction between PVA fibers and cement hydrates at their interface, thereby improving resistance to pull-out. This observation aligns with the inferences drawn from the flexural strength and behavior analysis results.

**Figure 14:**
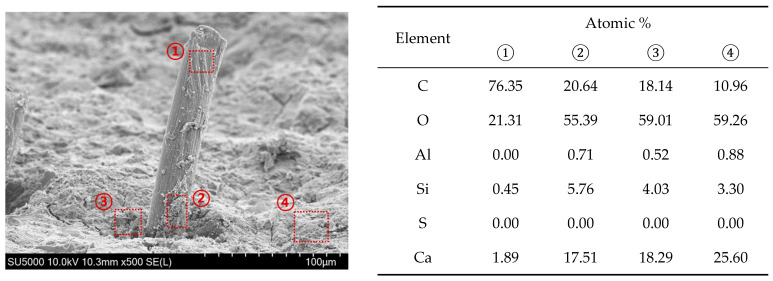
EDS analysis (P12-1-GO).

Figure 14 presents the results from the EDS analysis of the P12-1-GO mixture. Area 1 represents the fractured surface of the PVA fiber, primarily composed of carbon and oxygen. Area 2, located on the surface of the PVA fiber, contains cement hydration products, which are likely to be the amorphous morphology of C-S-H gel. Notably, this area demonstrates a carbon content exceeding 20%, indicating a significant presence of GO. Adjacent to the PVA fiber is Area 3, also identified as a C-S-H gel region. However, this area displays a relatively lower carbon content compared to Area 2. Area 4 is deduced to be a zone where C-S-H and Calcium Hydroxide (CH) coexist, with a recorded carbon content of 10.96%. The EDS results suggest a trend where both carbon and silicon concentrations diminish with increasing distance from the PVA fiber. Given the substantial role of GO in the formation of C-S-H, it can be inferred that GO is densely distributed around and on the surface of the PVA fibers within the hydrated cement matrix. This distribution leads to an increase in the quantity of C-S-H in these specific areas, thereby potentially impacting the flexural strength and behavioral characteristics of the composites.

## 4. Conclusions

In this study, the effects of incorporating GO and PVA fibers into cement mixtures on their rheological properties and flexural behaviors were experimentally analyzed. Ten cement mixtures, with and without 6 mm and 12 mm PVA fibers and 0.05% GO, were prepared. The change in shear stress was measured by varying the shear rate for each mixture. For the specimens from each mixture, flexural strength tests were conducted. Flexural strain analysis under load history was performed using the DIC technique. Additionally, the microstructures of each mixture were examined using SEM and EDS analyses. This led to the following conclusions.

- The inclusion of GO in cement mixtures increases static yield stress, while adding PVA fibers raises both static yield stress and critical shear strain, particularly with longer or more abundant fibers. Notably, the combined effect of PVA fibers and GO significantly increases yield stress more than either additive alone.
- Adding GO results in higher mixture viscosity, and combining it with PVA fibers leads to pronounced viscosity fluctuations, indicating a more significant effect on the rheological properties. Furthermore, the length of PVA fibers is more influential than the number of strands in determining the rheological characteristics of the mixtures.
- Adding GO to PVA fiber-reinforced cement mixtures reduces the crack propagation rate and delays crack initiation. While smaller amounts of PVA fibers lead to sudden failure after cracking, incorporating GO increases pre-crack strain by 30 to 50% and mitigates further crack propagation.
- SEM analysis shows that GO contributes to a denser microstructure and effectively interacts with PVA fibers, enhancing the adherence of hydration products at their interface. This improves resistance to pull-out and overall mechanical strength.
- EDS analysis reveals a concentrated presence of GO around and on PVA fibers, promoting increased C-S-H gel formation, crucial for the cement’s mechanical properties. This may be attributed to the functionalized oxygen groups in GO, which enhance the hydration process and play a vital role in improving the composite’s flexural strength and altering its behavior.

## Figures and Tables

**Table 1:** Chemical composition of OPC.

Component	CaO	SiO_2_	Al_2_O_3_	Fe_2_O_3_	SO_3_	IR ^a^	LOI ^b^
Portion (%)	64.90	21.49	4.21	3.50	0.70	0.65	-
ASTM C150 [21]	-	Min 20.0	Max 6.0	Max 6.0	Max 3.0	0.75	Max 3.0

^a^ Insoluble residue. ^b^ Loss on ignition.

**Table 2:** Properties of GO and PVA fiber.

Material	Property	Value
GO	Carbon	90–95%
	Oxygen	5–10%
	Surface area	200–300 m^2^/g
	Mean particle size	450 nm
	Thickness	~10 nm
	Specific gravity	1.91
	Bulk density	~1.8 g/cm^3^
PVA fiber	Diameter	26 μm
	Length	6.0 mm, 12.0 mm
	Tensile strength	1200 MPa
	Specific gravity	1.3 ± 0.1
	Elastic modulus	24.5 GPa

**Table 3:** Mix proportion and specimen IDs.

ID	w/c	GO (wt%)	PVA (vol%)	
			6 mm	12 mm
CTRL	0.5	-	-	-
GO	0.5	0.05	-	-
P6-1	0.5	-	1	-
P6-2	0.5	-	2	-
P12-1	0.5	-	-	1
P12-2	0.5	-	-	2
P6-1-GO	0.5	0.05	1	-
P6-2-GO	0.5	0.05	2	-
P12-1-GO	0.5	0.05	-	1
P12-2-GO	0.5	0.05	-	2

**Table 4:** Loads, deflections, and back-calculated elastic modulus of the mixtures.

ID	1st Peak		2nd Peak		E (GPa)
	Load (kN)	Deflection (mm)	Load (kN)	Deflection (mm)	
CTRL	1.563	0.028	-	-	18.25
GO	1.631	0.028	-	-	19.21
P6-1	1.965	0.033	1.331	0.253	19.49
P6-2	1.599	0.034	1.893	0.244	15.53
P12-1	1.718	0.039	1.562	0.234	14.43
P12-2	1.895	0.037	1.982	0.214	17.01
P6-1-GO	2.163	0.037	1.360	0.220	19.69
P6-2-GO	1.966	0.035	2.064	0.229	18.48
P12-1-GO	2.019	0.031	1.657	0.228	21.80
P12-2-GO	2.251	0.036	2.449	0.315	21.10

**Table 5:** Average strains near crack for the mixtures with PVA fibers and GO.

ID	ε_xx_ at 1st Peak (10^−3^)		ⓑ/ⓐ (%)	ε_xx_ at 2nd Peak (10^−3^)		ⓑ/ⓐ (%)
	Without GO (ⓐ)	With GO (ⓑ)		Without GO (ⓐ)	With GO (ⓑ)	
P6-1	2.77	4.17	150.54	95.55	88.41	92.53
P6-2	4.09	6.14	150.12	73.64	74.18	100.73
P12-1	4.26	5.52	129.58	80.46	82.31	102.30
P12-2	3.74	5.26	140.64	139.67	145.88	104.45

## Data Availability

Data are available from the authors upon reasonable request.
